# Inflammation and enhanced atherogenesis in the carotid artery with altered blood flow in an atherosclerosis‐resistant mouse strain

**DOI:** 10.14814/phy2.14829

**Published:** 2021-06-10

**Authors:** Jian Zhao, Chaoji Huangfu, Zhihui Chang, Wei Zhou, Andrew T. Grainger, Zhaoyu Liu, Weibin Shi

**Affiliations:** ^1^ Departments of Radiology & Medical Imaging University of Virginia Charlottesville VA USA; ^2^ Department of Radiology Shengjing Hospital of China Medical University Shenyang China; ^3^ Center for Disease Control and Prevention Western Theater Command Lanzhou China; ^4^ Department of Nephrology The Second Affiliated Hospital of Liaoning University of Traditional Chinese Medicine Shenyang China; ^5^ Biochemistry & Molecular Genetics University of Virginia Charlottesville Virginia USA

**Keywords:** atherosclerosis, blood flow, carotid artery, genetic background, hyperlipidemia

## Abstract

Ligation of the common carotid artery near its bifurcation in apolipoprotein E‐deficient (Apoe^−/−^) mice leads to rapid atherosclerosis development, which is affected by genetic backgrounds. BALB/cJ (BALB) mice are resistant to atherosclerosis, developing much smaller aortic lesions than C57BL/6 (B6) mice. In this study, we examined cellular events leading to lesion formation in carotid arteries with or without blood flow restriction of B6 and BALB Apoe^−/−^ mice. Blood flow was obstructed by ligating the left common carotid artery near its bifurcation in one group of mice, and other group received no surgical intervention. Without blood flow interruption, BALB‐Apoe^−/−^ mice formed much smaller atherosclerotic lesions than B6‐Apoe^−/−^ mice after 12 weeks of Western diet (3,325 ± 1,086 vs. 81,549 ± 9,983 µm^2^/section; *p* = 2.1E‐7). Lesions occurred at arterial bifurcations in both strains. When blood flow was obstructed, ligated carotid artery of both strains showed notable lipid deposition, inflammatory cell infiltration, and rapid plaque formation. Neutrophils and macrophages were observed in the arterial wall of BALB mice 3 days after ligation and 1 week after ligation in B6 mice. CD4 T cells were observed in intimal lesions of BALB but not B6 mice. By 4 weeks, both strains developed similar sizes of advanced lesions containing foam cells, smooth muscle cells, and neovessels. Atherosclerosis also occurred in straight regions of the contralateral common carotid artery where MCP‐1 was abundantly expressed in the intima of BALB mice. These findings indicate that the disturbed blood flow is more prominent than high fat diet in promoting inflammation and atherosclerosis in hyperlipidemic BALB mice.

## Graphical abstract

We examined cellular events leading to lesion formation in carotid arteries with or without blood flow restriction of C57BL/6 (B6) and BALB/c (BALB) Apoe^−/−^ mouse strains. Without blood flow interruption, BALB‐Apoe^−/−^ mice formed much smaller atherosclerotic lesions than B6‐Apoe^−/−^ mice after 12 weeks of Western diet. When blood flow was obstructed, the ligated carotid artery of both strains showed notable lipid deposition, inflammatory cell infiltration and rapid plaque formation.

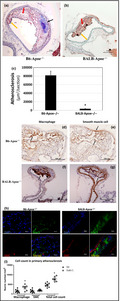

## 1 INTRODUCTION

Atherosclerosis in the carotid arteries is a major cause of ischemic stroke (Flaherty et al., 2013; Lovett et al., 2004), which accounts for over 80% of all stroke cases. Vulnerable atherosclerotic plaques that typically contain a large lipid core, a thin fibrous cap, and intraplaque hemorrhage in the carotid bifurcations are associated with future transient ischemic attacks and stroke (Mughal et al., 2011). Because of the difficulties in studying pathogenic mechanisms in humans, the mouse has emerged as the most valuable alternative for studying atherosclerosis and many other diseases. Wild‐type mouse strains do not develop atherosclerotic plaques beyond the aortic root even after prolonged dietary exposure to an atherogenic diet (Paigen et al.,^1987^, ^1990^), thus precluding their use for studying carotid atherosclerosis. When null in apolipoprotein E (Apoe^−/−^), mice develop spontaneous hyperlipidemia and atherosclerosis in the carotid arteries and other large and medium‐sized arteries (Nakashima et al., 1994; Rosenfeld et al., 2000). Atherosclerotic lesions of Apoe^−/−^ mice appear at branch points or curvatures of arteries and progress from the foam cell stage to the advanced stage with fibrous caps and necrotic areas, as seen in humans. Genetic backgrounds have a dramatic influence on the growth of atherosclerosis in the carotid arteries and other arterial sites of mice (Dansky et al., 1999; Shi et al., 2000; Zhao et al., 2019). For example, C57BL/6 (B6)‐Apoe^−/−^ mice developed large advanced lesions in the carotid arteries after 12 weeks of Western diet, while C3H‐Apoe^−/−^ mice developed no lesions (Zhao et al., 2019). Like C3H mice, BALB/cJ (BALB) mice are highly resistant to atherosclerosis with regard to lesion formation in the aortic root (Paigen et al., 1990; Tian et al., 2005). In F2 mice derived from BALB Apoe^−/−^ strains, we have mapped multiple loci for carotid atherosclerosis (Grainger et al., 2017; Rowlan et al., 2013). As the yield of loci is often dependent on disparity in the phenotype of choice between two progenitor strains from which F2 mice are bred (Moore & Nagle, 2000), we postulate that BALB mice are resistant to carotid atherosclerosis relative to B6 mice.

Atherosclerosis occurs preferentially at the place of large and medium arteries where there is low and oscillatory wall shear stress (Ku et al., 1985). When blood flow is partially or completely obstructed by ligation of the common carotid artery or its branches, atherosclerosis occurs in the straight region of the vessel in hyperlipidemic Apoe^−/−^ mice, with contributions from endothelial cells, smooth muscle cells, inflammatory cells, and blood lipids (Chang et al., 2017; Eschert et al., 2009; Nam et al., 2009; Zhao et al., 2019). Lesions in the ligated vessel rapidly progress from foam cell lesions to advanced plaques containing foam cells, fibrous cap, necrotic area, and neovessels, all of which occur within 4 weeks. Lesion progression in this model can be accelerated by feeding a high fat diet, as seen in hyperlipidemic mouse models of primary atherosclerosis (Liu et al., 2015; Shi et al., 2000; Tian et al., 2005). The easiness in reproducibly creating the mouse model has opened up a new path for atherosclerosis research with hyperlipidemic mice. Complete ligation of the common carotid artery of mice is a highly feasible, reproducible model in which disturbed blood flow initiates a rapid atherogenic process involving the arterial wall, inflammatory cells, and blood lipids. The extent of lesion development in this model depends on the genetic background and hyperlipidemia of mice (Chang et al., 2017; Zhao et al., 2019). In this study, we used two different models to characterize BALB‐Apoe^−/−^ mice for plaque formation in the carotid arteries with and without blood flow restriction by comparing with B6‐Apoe^−/−^ mice.

## 2 METHODS

### 2.1 Mice

B6‐Apoe^−/−^ mice purchased from the Jackson Laboratory were bred to generate mice used for this study. BALB‐Apoe^−/−^ mice were generated in our laboratory using the classical congenic breeding protocol (Tian et al., 2005). Mice were weaned at 3 weeks of age onto a chow diet, switched onto a Western diet which contained 21% fat, 48.5% carbohydrate, 17% protein, and 0.2% cholesterol (TD 88137, Envigo) 1 week before receiving a ligation of the left common carotid artery, and remained on the diet thereafter. To study primary atherosclerosis, mice received no surgical intervention to the carotid artery but were challenged with the Western diet, which started at 6 weeks of age and remained on the diet for 12 weeks. All animal‐related procedures were conducted in compliance with the NIH guide for the care and use of laboratory animals and approved by the Institutional Animal Care and Use Committee.

### 2.2 Ligation of the common carotid artery

The left common carotid artery of mice was ligated near its distal end as reported (Chang et al., 2017; Zhao et al., 2019). Briefly, 4–8 week‐old mice were anesthetized with a mixture of ketamine (80 mg/kg, Aveco Inc.) and xylazine (8 mg/kg, AnaSed, Lloyd Laboratories) as reported (Eschert et al., 2009; Zhao et al., 2019). A midline incision on the front of the neck was made to expose the left common carotid artery under a dissecting microscope and the artery was completely ligated with a 4–0 silk suture just below its bifurcation. The skin incision was then closed with a surgical glue.

### 2.3 Tissue preparation and lesion quantification

Mice were euthanized via a prolonged exposure to isoflurane inhalation 3 days, 1 or 4 weeks after the ligation of the left common carotid artery. The vasculature was flushed first with saline and then with 10% formalin via the left ventricle of the heart. The front soft tissues of the neck encompassing the left and right common carotid arteries were dissected out, embedded in OCT compound (Tissue‐Tek, Miles Inc.), and cross‐sectioned in 10‐µm thickness. Serial sections were collected and mounted on superfrost slides with 8 sections per slide. 3 evenly spaced slides were chosen for hematoxylin and eosin (H&E) staining for each mouse. 1 section on each stained slide was subject to morphometric measurements of the ligated left common carotid artery and the contralateral right common carotid artery. Luminal area and areas encircled by the internal and external elastic laminae were measured using the Zeiss AxioVision 4.8 software. Intimal lesion area and medial arterial wall area were calculated. Measurements made from 3 separate slides were averaged for each vessel and this average was used for statistical analysis as reported (Zhao et al., 2019).

For visualization of lipid deposition in arterial walls, selected sections were stained with oil red O as reported (Zhao et al., 2019). For evaluation of quantitative differences in lipid accumulation in arterial walls of two strains, sections from 3 B6 and 5 BALB mice that received 1 week of carotid ligation were stained with oil red O in the same batch. Red‐stained areas in medial arterial walls of ligated and contralateral carotid arteries were quantified using the red threshold function of ImageJ.

Primary atherosclerosis in the left common carotid artery bifurcation of both Apoe^−/−^ strains was measured, as previously reported (Li, Li, et al., 2008; Rowlan et al., 2013). Briefly, the distal common carotid artery and adjacent branches were dissected en bloc and embedded in OCT compound. 10‐µm‐thick cross sections were collected every three sections and stained with oil red O and hematoxylin. Lesion areas were quantified using the Zeiss AxioVision software. The lesion areas of 5 sections with the largest readings were averaged for each mouse and this average was used for statistical analysis.

### 2.4 Immunohistochemical analysis

Immunostaining was performed on frozen sections using the following primary antibodies: rat anti‐mouse Ly6G antibody (eBioscience), rat anti‐mouse CD4 antibody (Millipore), rat anti‐mouse macrophage/monocyte IgG, clone MOMA‐2 (Serotec), goat anti‐α‐smooth muscle actin antibody (Arigo Biolaboratories), rat anti‐mouse macrophage/monocyte IgG, clone M3/84(BD Biosciences), rabbit anti‐mouse CD8 IgG (D4W2Z, Cell Signaling), goat anti‐mouse IgG (MJE00B, R&D Systems), and polyclonal goat anti‐mouse N‐formyl peptide receptor (FPR) (Santa Cruz Biotech) and MCP‐1 antibodies (R&D System). Immunoreactivity was visualized with the Vectastain Elite ABC Kit (Vector Laboratories). Sections stained with Alexa Fluor 488‐conjugated goat anti‐rat IgG (H+L) (4416S, Cell Signaling), NL557‐conjugated donkey anti‐goat IgG (H+L) (NL001, R&D Systems), or DAPI fluoromount‐G (0100–20, Southern Biotech) were directly visualized by fluorescence microscopy.

The density of macrophages, smooth muscle cells, and total cells in primary atherosclerosis was quantified on immunofluorescent‐stained sections for three or five mice per strain. Cells in two different fields at ×40 magnification were counted for each section. Selected sections of ligated arteries were immunostained for inflammatory cells, smooth muscle cells, and MCP‐1.

### 2.5 Statistical analysis

Data were expressed as mean ± SE, with “n” indicating the number of mice. Student's *t*‐test was used to determine statistical significance for differences between groups in various measurements. Differences were considered statistically significant at *p* ≤ .05.

## 3 RESULTS

### 3.1 Primary atherosclerosis in the carotid artery

**FIGURE 1:**
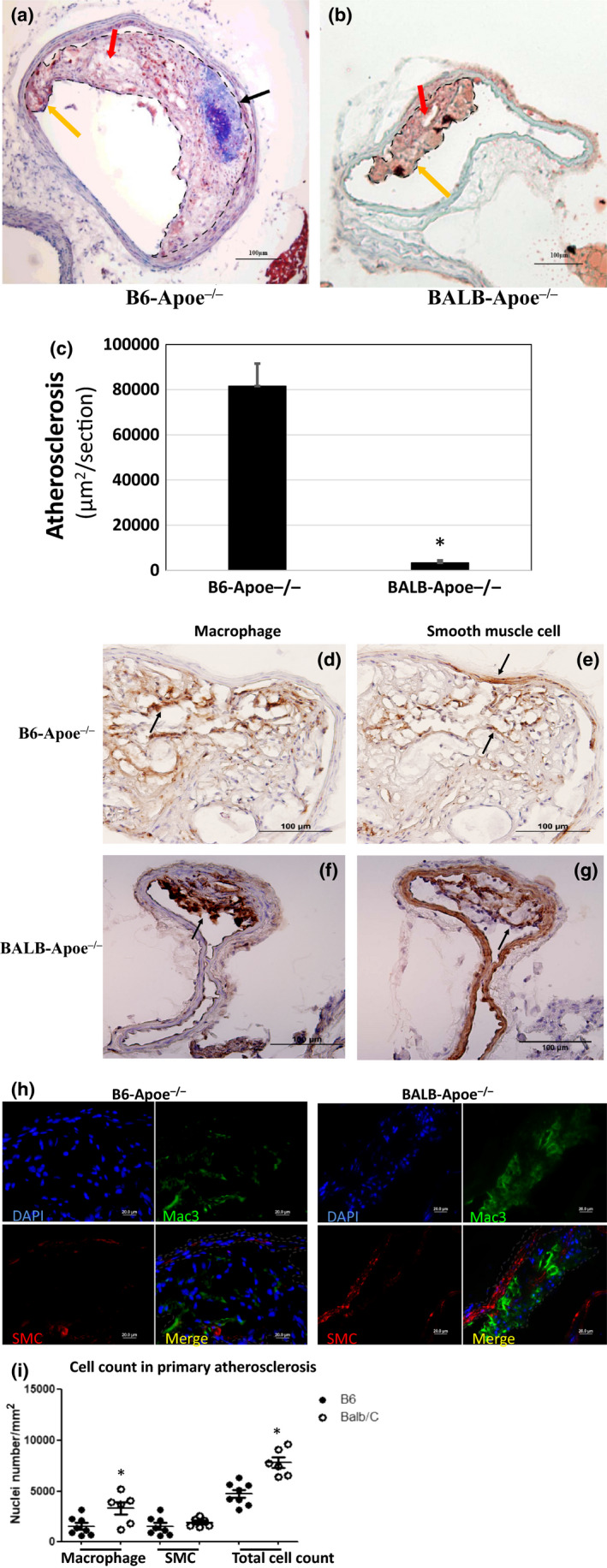
Primary atherosclerosis in the carotid artery of B6 and BALB Apoe^−/−^ mice fed 12 weeks of Western diet. (a and b) Oil red O‐stained sections of the left carotid artery of a B6 and a BALB mouse. B6 mouse developed an advanced lesion containing fatty, fibrous, necrotic, and calcium components, while BALB mouse developed a much smaller lesion. The lesion area is encircled by the dash line. Black arrow points at calcification (blue spot), red arrow at necrosis, yellow arrow at fat fibrous tissue. (c) Atherosclerotic lesion sizes in the left carotid artery of female B6 and BALB Apoe^−/−^ mice. Results are means ± SE of 12 (BALB) or 20 mice (B6). (d through g) Immunostaining against macrophages and smooth muscle cells in atherosclerotic lesions. Black arrow points at stained macrophages or smooth muscle cells. (h) Immunofluorescence staining for macrophages (green), smooth muscle cells (red), and nuclei (blue) in primary atherosclerotic lesions. (i) Quantitative analysis of the cell density of macrophages, smooth muscle cells, and total cells in atherosclerotic lesions. Values are means ± SE of cell count per mm^2^ in sections from three or four mice per group. Unpaired Student's *t*‐test was performed to compare differences between the two strains. *p < .05

Eccentric atherosclerotic lesions occurred at or near the bifurcation of the common carotid artery in both strains (Figure 1a,b). Compared to BALB, B6 mice developed more advanced atherosclerotic lesions containing calcification and numerous necrotic areas. The average lesion size of B6 mice (n = 20) was 81,549 ± 9,983 µm^2^/section, 25‐fold larger than the size of BALB mice (3,325 ± 1,085 µm^2^/section, *p* = 2.12E‐07, n = 12) (Figure 1c). Standard immunostaining showed the abundance of macrophages and smooth muscle cells in the lesions of both strains (Figure 1d–g). On immunofluorescent‐stained sections, the density of macrophages, smooth muscle cells, and nuclei in atherosclerotic lesions were quantified (Figure 1h). BALB mice had approximately a 3‐fold higher macrophage density than B6 mice (3,324 ± 613 vs. 1,208 ± 89 cells/mm^2^; *p* = .019) (Figure 1i). The density of total cells in the lesions was also significantly higher BALB mice (7,805 ± 537 vs. 4,731 ± 388 cells/mm^2^; *p* = .001). In contrast, the density of smooth muscle cells was comparable between the two strains (BALB: 1,906 ± 180; B6: 1,539 ± 321 cells/mm^2^; *p* = .34).

### 3.2 Lesion formation in ligated carotid artery

**FIGURE 2:**
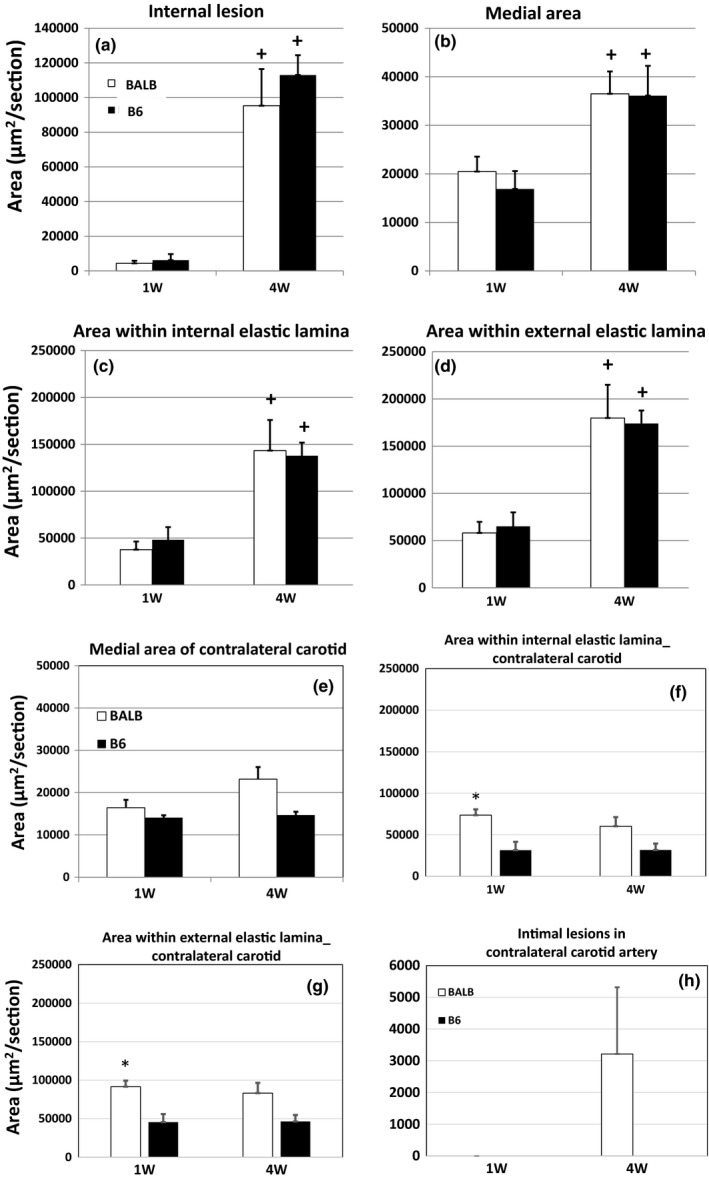
Morphometric measurements of the ligated left common carotid artery and contralateral right common carotid artery of B6 and BALB Apoe^−/−^ mice fed the Western diet. Intimal lesions (a), medial area (b), cross‐sectional areas encircled by the internal (c) and external elastic lamina (d) in the ligated carotid artery; medial area (e), cross‐sectional area encircled by the internal (f) or the external elastic lamina (g), and intimal lesions (h) of the contralateral common carotid artery. Measurements were made on the carotid arteries 1 (1 W) and 4 weeks (4 W) after ligation. Values are means ± SE of three to five mice per group. Unpaired Student's *t*‐test was performed to compare the difference between two groups or measures. *p < .05 versus B6 mice at the same time point, and ^+^ *p* < .05 versus 1 week in the same mouse strain

Morphometric measurements of the left common carotid artery after 1 and 4 weeks of ligation were compared between B6 and BALB Apoe^−/−^ mice. At 1 week, minimal intimal lesions formed in the vessel of either strain (B6: 6,157 ± 3,493, n = 3; BALB: 4,373 ± 1,456 µm^2^/section, n = 5) (Figure 2a). At 4 weeks, both strains developed substantial intimal lesions in the ligated arteries, with B6 having an average lesion size of 112,987 ± 11,395 µm^2^/section (n = 5) and BALB having a lesion size of 95,277 ± 21,139 µm^2^/section (n = 5). The lesion sizes were not significantly different between the two strains (*p* = .488).

The medial area of ligated carotid arteries was comparable between B6 and BALB mice at either 1 or 4 week post‐ligation time point (Figure 2b). A comparison between the two time points in measures showed a time‐dependent increase in the medial area of the ligated arteries for either strain (*p* < .05). The areas encircled by the external or internal elastic lamina of ligated arteries were comparable between the two strains at either 1 or 4 week post‐ligation time point (Figure 2c,d).

Comparison of measurements made at 1 and 4 weeks of post‐ligation time points showed a time‐dependent increase in the area encircled by either the external or the internal elastic lamina of ligated carotid arteries in both strains (Figure 2b–d), indicating the occurrence of positive vascular remodeling. In contrast, the contralateral common carotid artery of both strains did not show a time‐dependent increase in the area encircled by either the external or the internal elastic lamina (Figure 2e).

Compared to the contralateral carotid artery, the ligated artery showed a significant increase in medial area at 4 weeks for both strains and also for BALB at the 1‐week time point (Figure 2e). Medial areas of the contralateral carotid artery were comparable between the two strains at 1 week of ligation (B6: 14,075 ± 557; BALB: 16,424 ± 1,858 µm^2^/section) (Figure 2e). At 4 weeks, medial area of the contralateral carotid artery was larger in BALB than B6 mice (23,173 ± 2,846 vs. 14,684 ± 778 µm^2^/section) though the difference was not statistically significant (*p* = .087).

For the contralateral carotid artery, the area encircled by either the internal or the external elastic lamina was significantly larger in BALB than B6 mice at 1 week but not 4 weeks of ligation (Figure 2f,g). In addition, intimal lesions were observed in the contralateral carotid artery of 3 BALB mice but none of B6 mice 4 weeks after ligation (Figure 2h).

**FIGURE 3:**
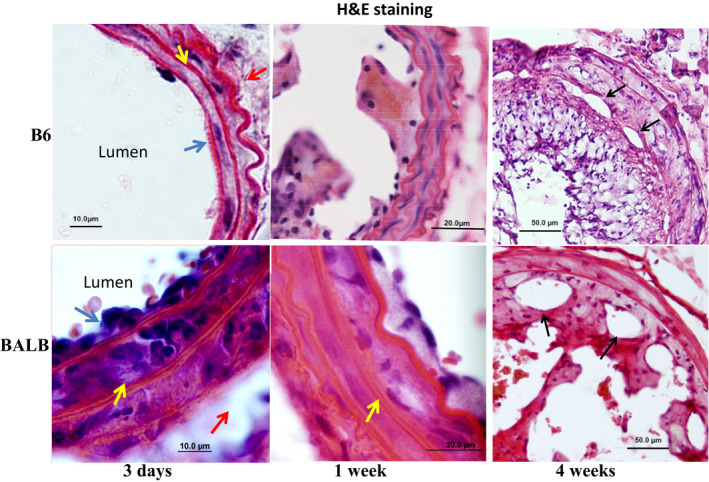
Histological analysis of intimal lesion formation in ligated common carotid arteries of B6 and BALB Apoe^−/−^ mice 3 days, 1 and 4 weeks after ligation. Sections were stained with the standard H&E method. The top row shows sections of the arteries from B6‐Apoe^−/−^ mice, and the bottom row shows sections of BALB‐Apoe^−/−^ mice. Blue arrows point at the intima, yellow arrows at the medial layer, red arrows at the adventitia of ligated artery, and black arrows point at neovessels. Infiltration of inflammatory cells in the medial layer of ligated carotid artery was observed in BALB but not B6 mice at 3 days and 1 week of ligation. Original magnifications: ×25 or ×40

Histological analysis showed the infiltration of inflammatory cells in the intima and medial layer of ligated arteries in BALB mice at 3 days after ligation, the earliest time point examined (Figure 3). In contrast, fewer inflammatory cells were observed in the intima and the medial layer of ligated arteries in B6 mice. At 1 week, intimal lesions were observable in ligated arteries of both strains, consisting of single or a few layers of foam cells. Foam cells were also observable in the medial layer of ligated arteries of BALB but not B6 mice. By 4 weeks, intimal lesions of both strains were large and contained neovessels. The medial arterial wall of B6 mice consisted of two layers of smooth muscle cells with each containing a spindle‐shaped, blue‐stained nucleus at the early time points of ligation (3 days, 1 week) but became thicker, more cellular and disorientated at the 4‐week time point. In contrast, the medial wall of BALB mice showed loss of the spindle‐shaped morphology of smooth muscle cells at all three time points examined.

**FIGURE 4:**
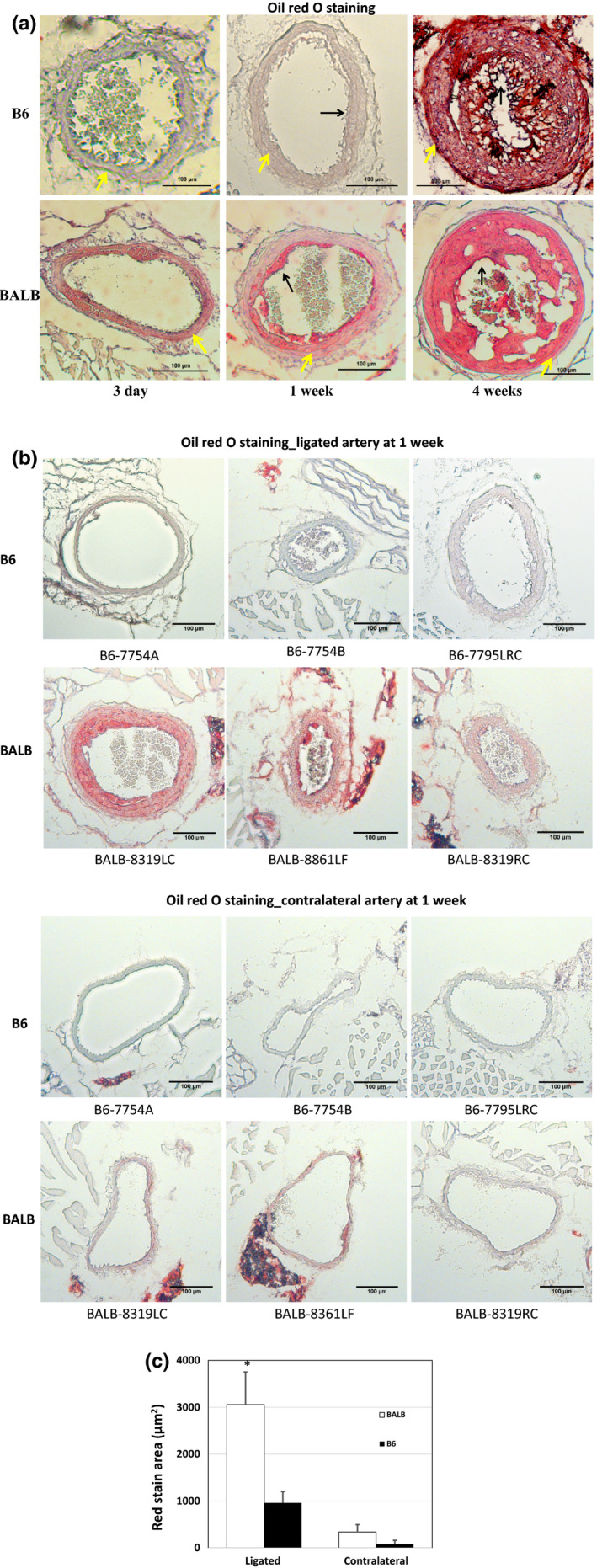
Oil red O‐stained sections of ligated common carotid arteries of B6 and BALB Apoe^−/−^ mice fed a Western diet. (a) Oil red O staining of ligated common carotid arteries of B6 and BALB mice with different durations of ligation. Intimal lesions are stained intensely red. The difference in red intensity for the medial layer of ligated arteries of the two strains at 3 days and 1 week of ligation was prominent. Blue arrows point at the intima, and yellow arrows at the medial layer. (b) Sections stained with Oil red O in the same batch of ligated carotid artery and contralateral carotid artery of B6 and BALB mice that received 1 week of carotid artery ligation. Each section represents an individual mouse. Original magnification: ×10. (c) Red areas in the medial wall of ligated and contralateral carotid arteries measured using the red threshold function of ImageJ. Results are means ± SE of three to five mice. *p* < .05

The intimal lesions in ligated carotid arteries were stained intensely red with oil red O (Figure 4a), indicating richness in lipids. The medial wall of ligated arteries was more intensely stained in BALB than B6 mice at earlier time points of ligation. By 4 weeks, the medial arterial walls of both strains were intensely stained. Stain intensity of tissue sections may vary from batch to batch. To minimize potential influence from the stain procedure, sections from B6 and BALB mice that received 1 week of carotid ligation were stained at the same time. Stronger staining of ligated carotid artery of BALB mice was grossly noticeable compared to B6 mice (Figure 4b). The contralateral carotid artery of BALB mice showed mild staining with oil red O, but the artery of B6 mice had no staining. The red‐stained areas of medial arterial walls were measured using the threshold function of ImageJ. Red areas of the ligated artery were 3‐fold larger in BALB mice than in B6 mice (3,055 ± 695 vs. 959 ± 243 µm^2^; *p* = .01) (Figure 4c). The contralateral artery also showed larger red‐stained areas in the medial layer of BALB mice (339 ± 157 vs. 80 ± 80 µm^2^) though the difference between the two strains did not reach statistical significance (*p* = .16).

### 3.3 Cytological analysis of lesion progression in the ligated carotid artery

**FIGURE 5:**
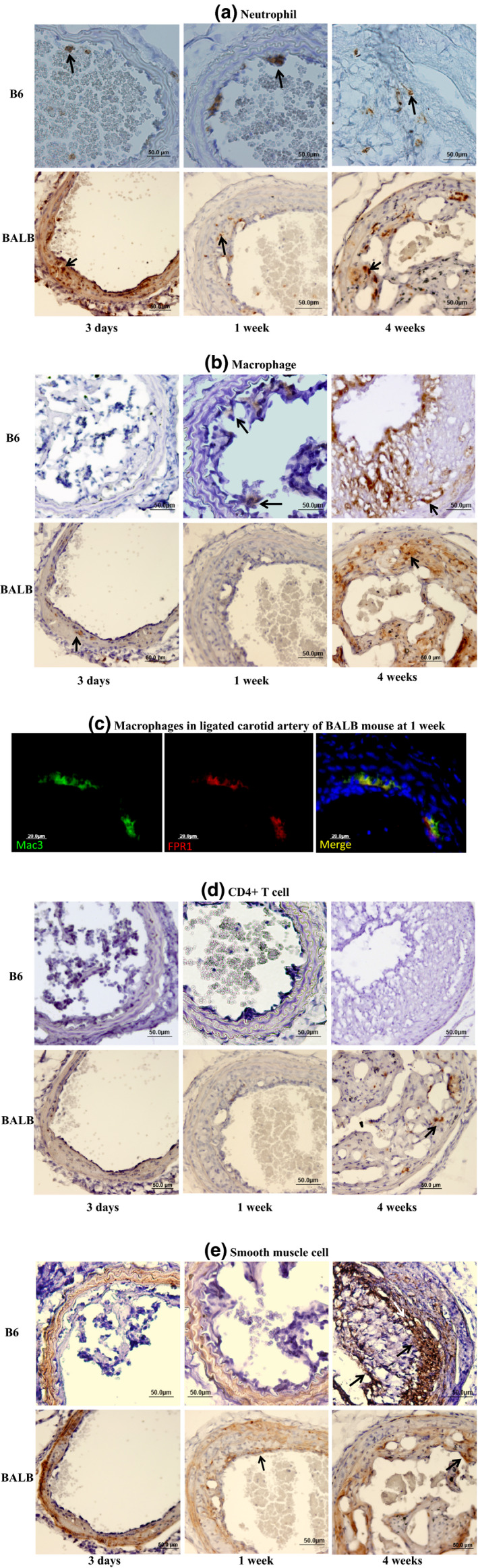
Immunohistochemical analysis of lesion formation in ligated carotid arteries of B6 and BALB Apoe^−/−^ mice with different durations of ligation. Sections were stained with antibodies against neutrophils (a), macrophages (b, c), CD4+ T cells (d), or smooth muscle cells (e). Arrows point at stained cells. Original magnification: ×40

At the earlier time points examined (3 days and 1 week), sporadic neutrophil adhesion to the intima of ligated arteries was observed in B6 mice, while in BALB mice neutrophils were seen infiltrated into the arterial walls and intimal lesions (Figure 5a). At 4 weeks, neutrophils were observed in intimal lesions of both strains although BALB mice had much more (18 vs. 8 cells in the scope under ×40 magnification).

Macrophages were observed in intimal lesions of both strains and also in the medial arterial wall of BALB mice at 3 days after ligation (Figure 5b). Fluorescent conjugated antibodies targeting Mac3 and FPR1 antigens were further applied to confirm the presence of macrophages in the intimal lesions (Figure 5c). At 4 weeks, both strains showed approximately 40% of the lesion area that strained for macrophages.

CD4+ T cells were observed in intimal lesions of BALB but not B6 mice (Figure 5d). CD8+ T cells were not detectable in the lesions of either strain (data not shown).

Immunoreactivity to smooth muscle α‐actin was detectable in intimal lesions of BALB mice as early as 1 week after ligation (Figure 5e). By 4 weeks, staining for α‐actin was obvious, accounting for 32%–36% of the intimal lesion areas of both strains. For B6 mice, staining was seen in the fibrous cap and deep portion of intimal lesions, while it was diffusive for BALB mice. A decline in α‐actin immunoreactivity was noticeable in the medial wall of ligated arteries for both strains, especially at 1 and 4 weeks after ligation.

### 3.4 Atherosclerosis in contralateral control carotid artery

**FIGURE 6:**
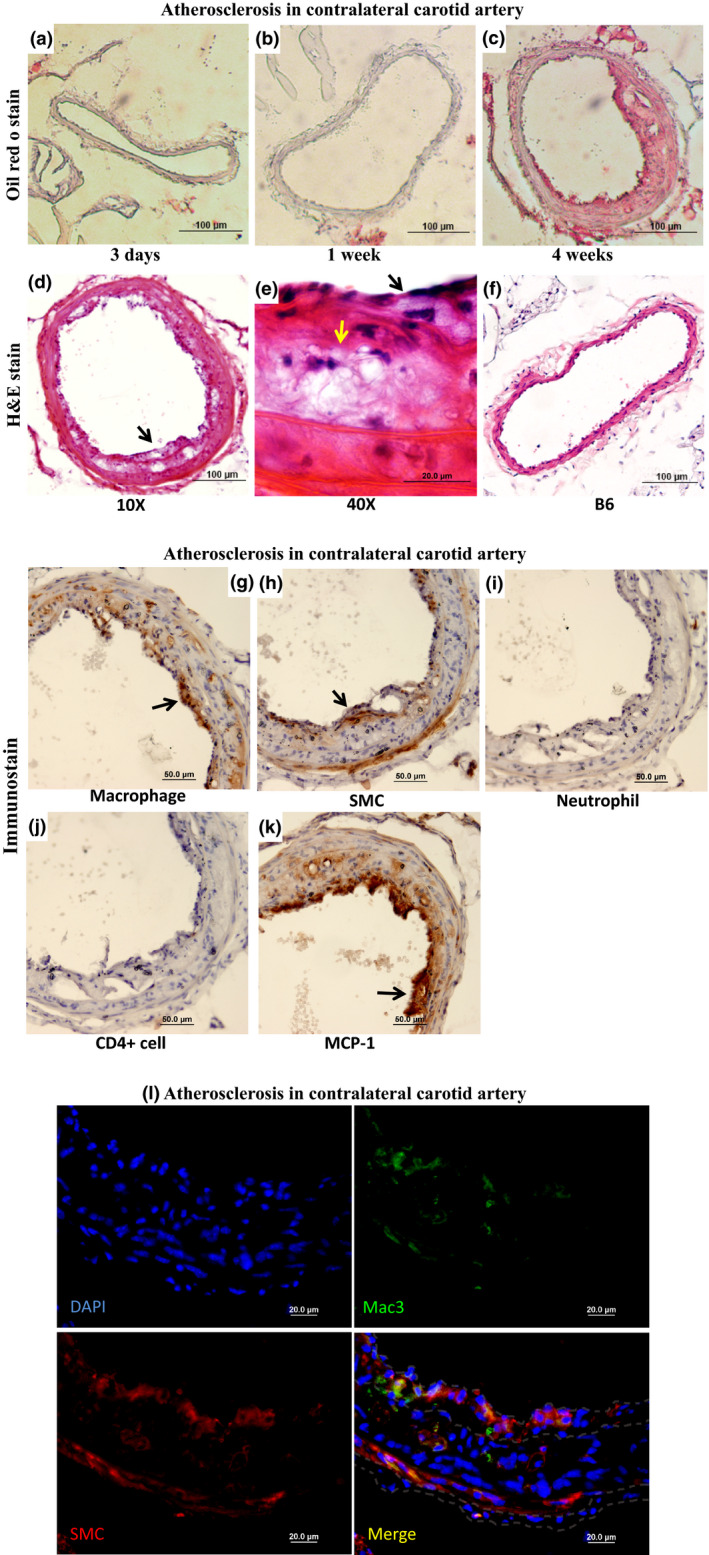
Atherosclerosis in the straight portion of contralateral common carotid artery in BALB‐Apoe^−/−^ mice that received left common carotid artery ligation. (a, b, c) Oil red O‐stained sections of the contralateral carotid artery of BALB mice 3 days, 1 and 4 weeks after ligation; (d, e, f) H&E‐stained sections from BALB (d, e) and B6 (f) mice 4 weeks after ligation; (g through k) Immunostaining with antibodies against macrophages, smooth muscle cells (SMC), neutrophils, CD4+ cells, or MCP‐1. Arrows point at foam cells or stained cells. (l) Immunofluorescence staining for macrophages (green), smooth muscle cells (red), and nuclei (blue)

There was no lesion in the contralateral right common carotid artery of BALB mice at earlier time points of ligation (Figure 6a,b). Atherosclerotic lesions occurred in the straight portion of the contralateral carotid artery in three of the five BALB mice 4 weeks after ligation. The average lesion size was 3,123 ± 2,103 µm^2^/section (n = 5; Figure 2h). Eccentric intimal lesions and adjacent medial walls stained intensely with oil red O (Figure 6c). H&E stain showed the presence of lipid‐laden foam cells and a more cellular cap in intimal lesions (Figure 6d,e). No lesion was found in the contralateral carotid artery of B6‐Apoe^−/−^ mice (n = 5, Figure 6f). Immunostaining further demonstrated the existence of macrophages and smooth muscle cells in the lesions (Figure 6g,h). Both types of cells were abundant in regions of the fibrous cap. Neutrophils and CD4+ cells were undetectable in the intimal lesions or the arterial walls (Figure 6i,j). MCP‐1 was expressed in the intima, especially abundant near the luminal surface (Figure 6k). Immunofluorescence staining confirmed the abundance and distribution of macrophages and smooth muscle cells along the fibrous cap (Figure 6l).

## 4 DISCUSSION

In this study, we examined atherosclerotic lesion formation in the carotid artery of BALB‐Apoe^−/−^ mice through comparison with B6‐Apoe^−/−^ mice. Several interesting observations were made: First, BALB mice were highly resistant to carotid atherosclerosis, developing much smaller lesions in the vessel than B6 mice. Second, BALB mice developed as large carotid lesions as B6 mice when blood flow was obstructed. Finally, BALB but not B6 mice developed atherosclerosis in the straight portion of the contralateral carotid artery after one common carotid artery was ligated.

We observed a striking difference between B6 and BALB Apoe^−/−^ mice in the development of carotid atherosclerosis. On the Western diet, both Apoe^−/−^ strains developed severe hypercholesterolemia, but BALB mice had a higher level of HDL cholesterol (Tian et al., 2005), which protects against atherosclerosis. Circulatory levels of proinflammatory molecules such as P‐selectin and VCAM‐1, which accelerate atherosclerosis progression, were lower in BALB than B6 mice (Tian et al., 2005). On the Western diet, B6‐Apoe^−/−^ but not BALB‐Apoe^−/−^ mice develop type 2 diabetes (Liu et al., 2015; Li et al., 2011), a major risk for atherosclerosis. Local factors, particularly hemodynamic forces (VanderLaan et al., 2004), may also contribute to the variation in plaque formation. Indeed, the two Apoe^−/−^ strains exhibited a 25‐fold difference in carotid lesion sizes but only a 3.2‐fold difference in aortic lesion sizes after 12 weeks of Western diet (Tian et al., 2005). Blood flow in the aortic sinus is highly unsteady and three‐dimensional compared to the flow in the carotid bifurcation (Back et al., 2013), which may overwhelm genetic influences on plaque formation.

BALB Apoe^−/−^ mice showed accelerated atherosclerosis progression in the carotid artery when its blood flow was obstructed. Lipid deposition in arterial walls, an initial step in atherogenesis, was more prominent in BALB than B6 mice based on oil red O stain (Figure 4). Infiltration of inflammatory cells, including neutrophils and macrophages in medial arterial walls was more obvious in BALB mice at the early stages of lesion formation. Endothelial cells of BALB mice have an increased ability to oxidize LDL relative to cells of B6 mice though they show similar responses to oxidized LDL in term of inflammatory gene induction (Miyoshi et al., 2007). Thus, the increases in lipid deposition and LDL peroxidation may contribute to the early enhancement in inflammatory cell infiltration in the ligated artery of BALB mice. Foam cells, the hallmark of atherosclerosis, were observed in intimal lesions on H&E‐stained sections. Immunostaining demonstrated the presence of macrophages and smooth muscle cells. As the MOMA‐2 antibody is less effective in detecting marginal zone macrophages, antibodies against FPR1 and Mac3, which are specifically expressed by macrophages (Khazen et al., 2005; Zhang et al., 2015), were used to confirm the presence of these cells in smaller lesions. Smooth muscle cells are a cellular marker for the advance of atherosclerotic lesions, and these cells were observed in larger lesions of both strains and smaller intimal lesions near the luminal surface of BALB mice. Intraplaque neovascularization is considered a major feature of advanced atherosclerotic plaques (Doyle & Caplice, 2007). H&E staining clearly revealed the presence of intraplaque neovessels in both Apoe^−/−^ strains. In contrast, neovessels have barely been seen in primary atherosclerotic lesions of Apoe^−/−^ or other mouse model of atherosclerosis (Rosenfeld et al., 2000; Vries & Quax, 2016).

Compared to the carotid arteries of Apoe^−/−^ mice that received no surgical manipulation, the ligated vessels of both strains showed more lipid deposition in the arterial wall (Figures 1 and 4). A recent study has shown that endothelial glycocalyx integrity of partially ligated carotid artery of B6‐Apoe^−/−^ mice is compromised more by disturbed blood flow than by exposure to a Western diet (Mitra et al., 2018). Increased endothelial permeability would result in more ApoB‐containing lipoprotein accumulation in the walls of ligated arteries (Brown et al., 2004).

The Apoe^−/−^ mice were 4–8 weeks old when the carotid ligation surgery was performed. These life phases of mice correspond to teenage or young adult in humans. Fatty streaks become increasingly prevalent and some progress to advanced stages of atherosclerosis in children and young adults (Napoli et al., 2006). We previously have observed that age has little influence on fatty streak growth in mice (Li, Gilbert, et al., 2008). More importantly, the two Apoe^−/−^ strains compared had the same or similar ages for the current study.

A previous study showed that wild‐type BALB mice developed an 8‐fold larger intimal lesion in ligated carotid arteries than B6 mice 4 weeks after ligation though absolute lesion sizes were small in both strains (Harmon et al., 2000). In contrast, BALB‐Apoe^−/−^ mice developed similar sizes of lesions in ligated arteries as B6‐Apoe^−/−^ mice. The intimal lesions of wild‐type mice comprise smooth muscle cells whose replication determines intimal lesion sizes (Kumar & Lindner, 1997). In the Apoe^−/−^ model, intimal lesions contain not only smooth muscle cells but also macrophages and other inflammatory cells.

In the wild‐type model, the ligated carotid artery of B6 and BALB mice showed significant negative remodeling (Harmon et al., 2000). In contrast, both Apoe^−/−^ strains displayed significant positive remodeling of the ligated artery with regard to the time‐dependent enlargement of areas encircled by the external and the internal elastic lamina (Figure 2). Positive vascular remodeling has been observed in human coronary arteries with plaques (Glagov et al., 1987) and animal models of atherosclerosis (Bonthu et al., 1997; Zhao et al., 2019). The discrepancy between the wild‐type and Apoe^−/−^ models in vascular remodeling may be attributable to different lesion components in the ligated arteries. Smooth muscle cells are the major cellular component of intimal lesions in the ligated artery of wild‐type (Kumar & Lindner, 1997), while the intimal lesion of Apoe^−/−^ mice also contains macrophages and neutrophils. Macrophages and other inflammatory cells in the intimal lesions of Apoe^−/−^ mice produce MMP‐9, MMP‐12, and other enzymes that degrade extracellular matrix (Shi et al., 2003), while smooth muscle cells and their synthesized collagen in the lesion of wild‐type restrict vessel distension.

An interesting finding of this study is the occurrence of atherosclerosis in the straight portion of the contralateral carotid artery in atherosclerosis‐resistant BALB‐Apoe^−/−^ mice but not in susceptible B6‐Apoe^−/−^ mice. The ligation of one carotid artery causes increased blood flow through the contralateral carotid artery. Either elevated or decreased blood flow increases the infiltration of macrophages in the arterial wall (Bakker et al., 2008). We observed abundant MCP‐1 expression in the intima of the contralateral carotid artery in BALB mice. On the Western diet, BALB‐Apoe^−/−^ mice had higher non‐HDL cholesterol and triglyceride levels than B6‐Apoe^−/−^ mice (Supplementary Data), which could also accelerate lesion formation in the contralateral artery.

In summary, we have gained valuable insight from our present study of two Apoe^−/−^ strains concerning the complex interplay among genetic factors, hemodynamic force, and inflammation during the development of carotid atherosclerosis. We found that BALB mice were highly resistant to carotid atherosclerosis, but became susceptible when blood flow was altered. The finding that atherosclerosis occurred in the straight portion of the contralateral common carotid artery in BALB‐Apoe^−/−^ mice supports the concept that either low or high endothelial shear stress can be synergistic with hypercholesterolemia in promoting atherosclerosis (Koskinas et al., 2013). This also is the case for humans as well as animals (Singh & Tubbs, 2018).

## CONFLICT OF INTEREST

The authors declared they have no conflict of interest.

## AUTHOR CONTRIBUTIONS

JZ, CH, ZC, WZ, ATG, and WS designed and conducted the research; JZ and WS analyzed the data and drafted the manuscript; ZL and WS supervised the research. All authors approved the final version of the manuscript.

## Supporting information



Supplementary Material
